# War Handkerchiefs

**Published:** 1882-06

**Authors:** 


### WAR HANDKERCHIEFS.

The ancient custom of illustrating pocket handkerchiefs for the amusement and instruction of children has been seriously emulated by the French War Office for the benefit of the national army. The cotton handkerchiefs provided for the French soldiers are now decorated with special texts and cut for the technical and sanitary instruction of the wearers. The centre is occupied with the Cross of the Legion of Honor upon a red background, and the inscription underneath it, *Honneur et*

*Patrie.* Around this central point are grouped a circle of medallions, containing representations of officers of all grades, from the modest sub-lieutenant to the proud commandant of a *corps d'armée.* The different uniforms are pictured so distinctly that the French private can tell at a glance to what grade any officer whom he sees may have attained. The special pocket handkerchief prepared for the infantry soldier has exact drawings of the arms used by him, with explanations of their mechanism. The borders of the handkerchiefs are hemmed in with a framework of the national colors, and within the framework are printed a number of sanitary precepts to be observed on march and during a campaign. Here are some of the marching advices: "Wear the cravat loose. A strip of flannel day and night around the body in order to keep off the diarrhœa. Quench thirst with very small doses of wine, coffee, vinegar-and-water, or brandy-and-water. Take a piece of bread and a little coffee before the march. Spirituous drinks do more harm than good. Drink water neither hastily nor too cold. In quarters wash face and hands, and when possible the whole body. Wash the feet and rub in a little fat or brandy. Next cook the soup, and do it at once, even though feeling quite tired out."—*Scientific American.*

